# Authors from all over the world share their tech in *HardwareX* to battle COVID-19

**DOI:** 10.1016/j.ohx.2021.e00190

**Published:** 2021-03-09

**Authors:** Joshua M. Pearce

**Affiliations:** Richard Witte Professor of Materials Science & Engineering and Electrical & Computer Engineering, Director: Michigan Tech Open Sustainability Technology (MOST) Lab, Michigan Technological University, Houghton, MI 49931, Visiting Professor Équipe de Recherche sur les Processus Innovatifs (ERPI), Université de Lorraine, France, Visiting Professor of Photovoltaics and Nanoengineering, School of Electrical Engineering, Aalto University, Finland

**Figure:**
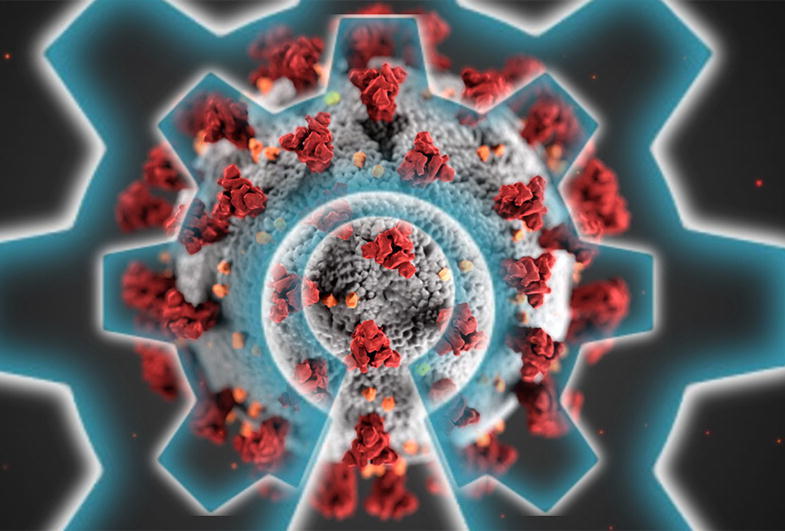

As COVID-19 continues to take a horrible toll on human lives, governments have been forced to take increasingly drastic measures to slow the disease in order to prevent our medical infrastructure from being overwhelmed. COVID-19 require hospitalization of 2.5% of young adults, but over 12% for retirees at minimum and they often need intense care. If COVID-19 infections are allowed to proceed unchecked there is not be enough medical hardware available to prevent the higher mortality rates observed in Italy in the beginning of the pandemic. At the same time our global supply chains were disrupted. There is thus a serious need for readily accessible medical hardware to help treat COVID-19 patients and to prevent more infections for the pandemic and future pandemics.

Today, with the evolution of digital manufacturing technologies such as 3-D printers and circuit milling systems, we can share designs with others who can then replicate medical-grade devices and products for the cost of locally-sourced materials. We have known about this approach of distributed manufacturing of high-quality equipment in the sciences for some time. *HardwareX*, specifically serves this function as an open access journal dedicated to publishing free and open hardware designs to benefit science. More often than not these technologies can be made with digital distributed manufacturing tools. Today as the pandemic still claims far too many lives throughout the world, large groups of makers, engineers, scientists and medical professionals are collaborating on the web to make *open source medical devices*, such as *ventilators*, to have a fast and easy solution that can be reproduced and assembled locally worldwide.

It will probably not surprise you to know that during the pandemic, we noticed that one of our *HardwareX* articles on a *programmable and low-cost ultraviolet room disinfection device*, became one of our most popular downloaded articles. It clearly was being used a lot more than in the past – with good reason. Dr. Todd Duncombe, my co-editor-in-chief pointed out that, “this is a great example of a tool used in a scenario where infection is a risk. There’s already been a lot of interest, as clinicians have to disinfect spaces between uses. The design here would have to be modified, as it’s currently for bacterial disinfection, not viral, but that’s exactly the kind of iterating and improving that *HardwareX* enables.”

**Figure:**
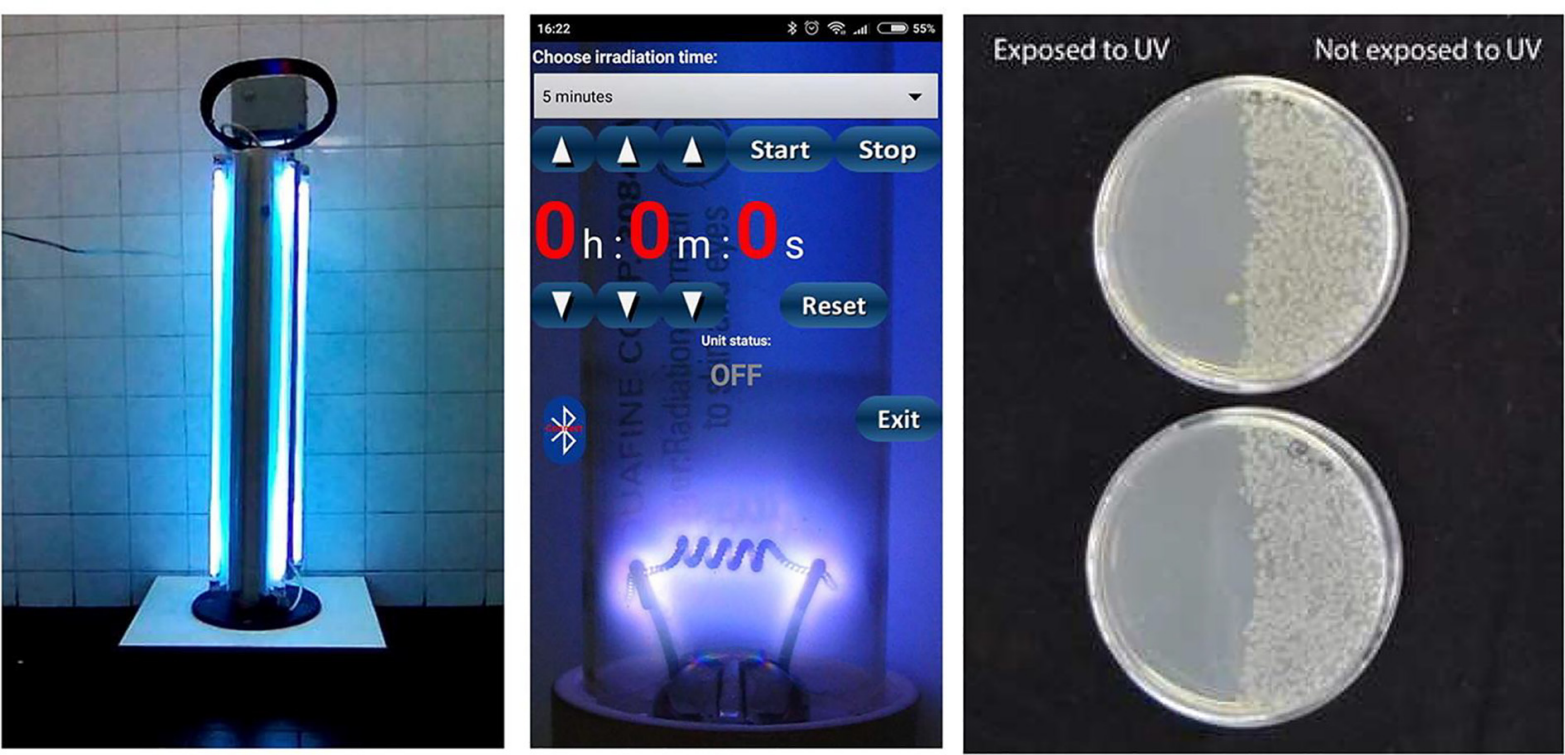

To help speed validation and dissemination of these open source technologies we sponsored a *special issue is dedicated to vetting the technical specifications and reproducibility of open medical hardware that can help during this global pandemic*. Elsevier, generously waived the APCs for all authors so that vital information could be spread as fast and as far as possible. The *HardwareX* Special Issue was an enormous success, despite running over a time period when many researchers could not access their labs because of lockdowns or quarantines. In addition, the same issues that made the need for distributed medical hardware so urgent – broken supply chains and extended shipping times, also challenged researchers to be able to both fabricate and test their technologies. Many *HardwareX* authors scattered throughout the globe were able to overcome these challenges and developed and tested medical hardware to battle the pandemic using methods that others could replicate during a pandemic.

Building on the room disinfection device that galvanized the idea, Marcel Bentancor and is team from Uruguay published the designs for *LUCIA: An open source device for disinfection of N95 masks using UV-C radiation*. The effectiveness of the device was demonstrated against an enveloped RNA virus, characteristics shared with the virus that causes COVID19, being capable of reducing the viral load by 4 orders of magnitude.

**Figure:**
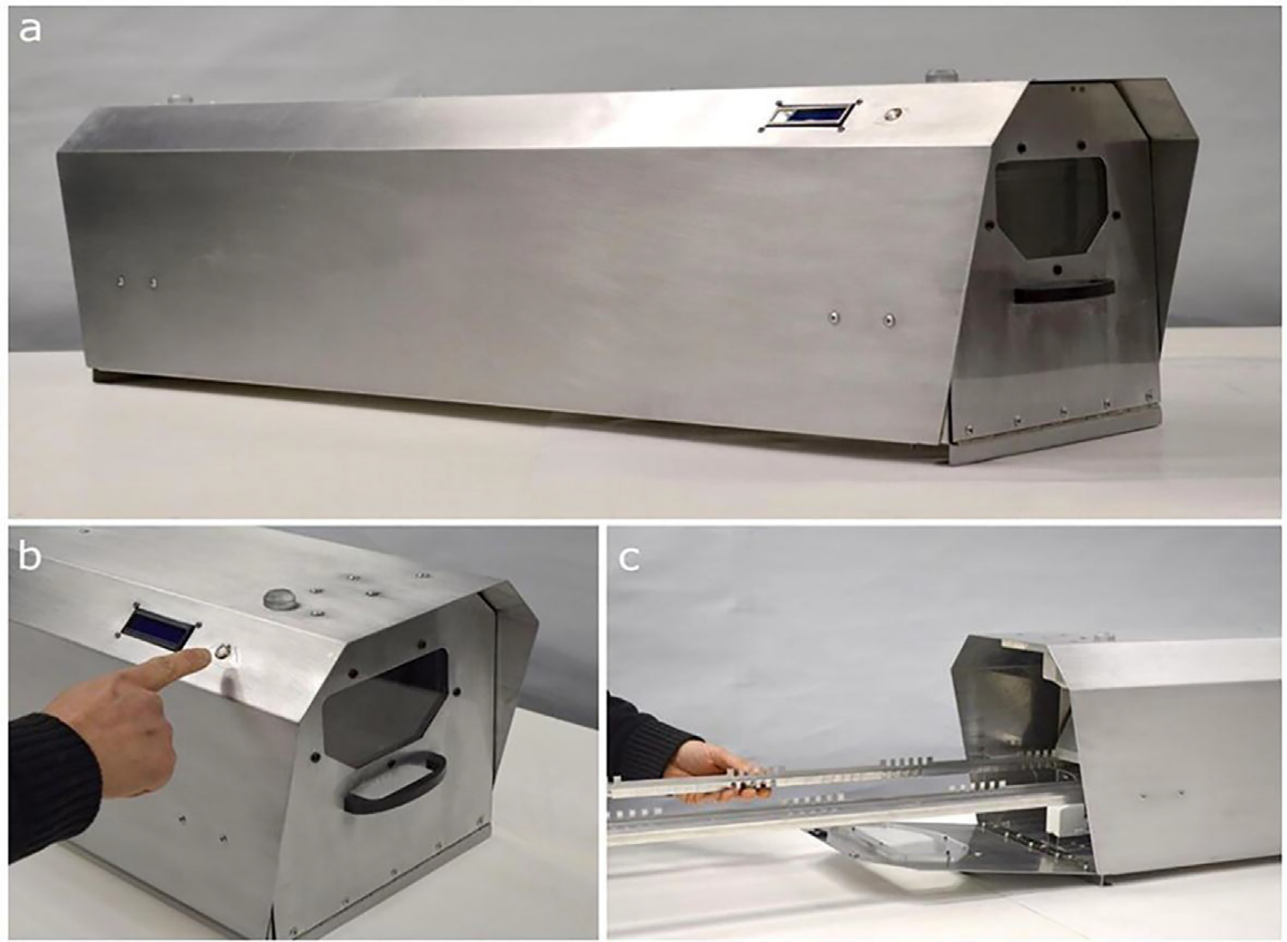

In the United States for a time last year even getting N95 masks was nearly impossible. To overcome this challenge, Nicholson et al. *modified a full-face snorkel mask to act as COVID-19 personal protective equipment* and performed quantitative testing on various full-faced snorkel masks with 3-D printed adapters that accept commercially available particulate filters. They shared a design that passed OSHA full-facepiece respirator standards. Similarly, it is possible to convert a self-contained breathing apparatus mask normally used by fire fighters (first responders also often did not have access to masks) to an *open source powered air-purifying particulate respirator* (PAPR). The open source PAPR has controllable air flow and enables breathing even if fan is disconnected or battery dies. It was tested for air flow as a function of battery life and was found to meet NIOSH air flow requirements for 4 h and 40 min, which is 300% over regular use. This device also used 3-D printing for the custom parts and commercially-available filters. At some points in the pandemic the shortage of respirators was so bad some medical doctors developed a 3-D printable mask that you could cut up a normal N95 to use as filter materials to make more masks. Several versions of this device were shared on the *NIH’s 3D Printing Exchange*. These 3-D printed parts need to be disinfected, however, as well. Skrzypczak et al., developed *an open source high-temperature RepRap for 3-D printing heat-sterilizable personal protective equipment (PPE)*. Normally high-temperature printers are only found in major manufacturing plants due to their high costs. The open source 3-D printer could be built for under $1000 in parts and successfully prints polyetherketoneketone (PEKK) and polyetherimide (PEI, ULTEM). Open source face masks were 3-D printed in PEKK and shown not to warp upon widely home-accessible oven-based sterilization. PPE printed with it could thus be thermally disinfected in your oven without worrying about melting.

**Figure:**
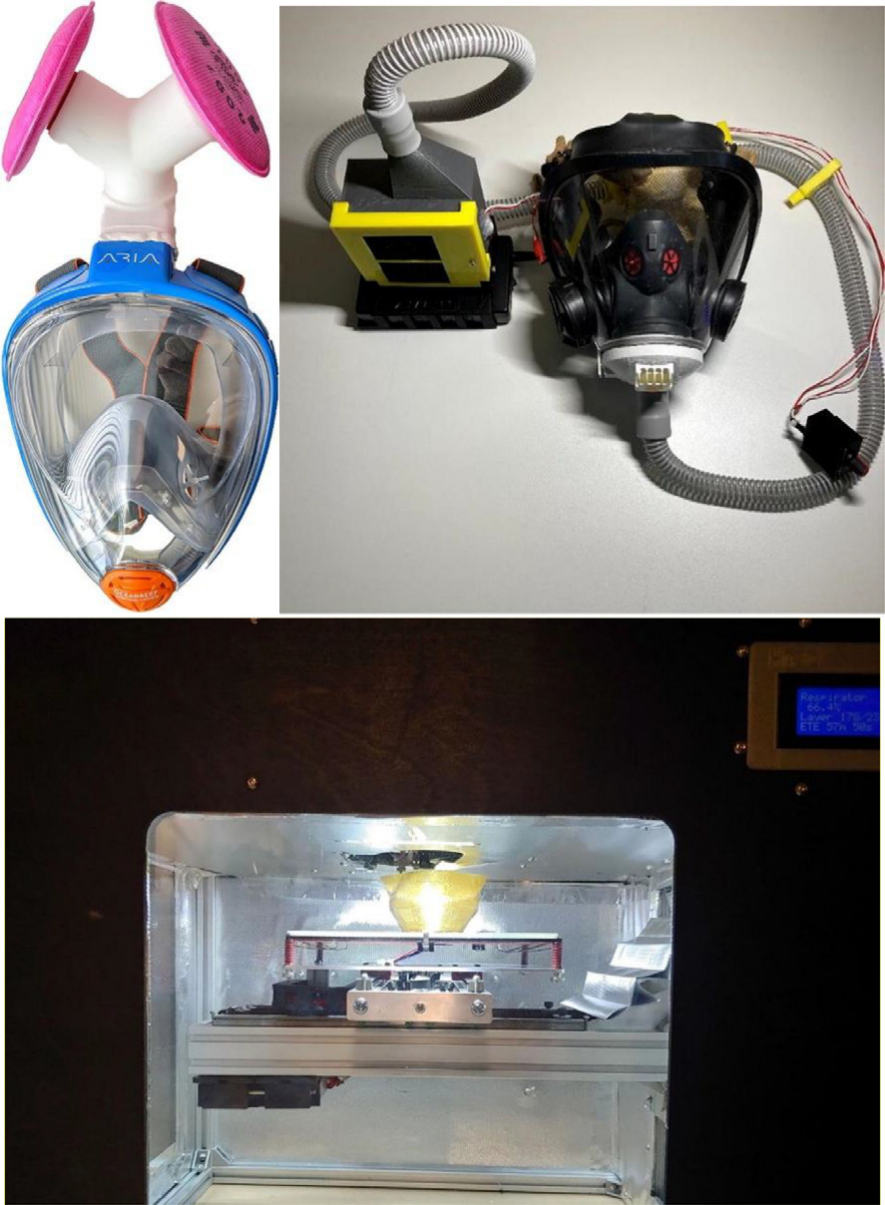

Addressing the same challenge, a collaboration between researchers in Sweden, Spain and the UK developed a *Pressure Optimized PowEred Respirator* (PROPER), which is a miniaturized wearable cleanroom and biosafety system for aerially transmitted viral infections such as COVID-19. A PROPER offers better protection than an N95 respirator mask, mainly because it is insensitive to seal fit and it shields the eyes as well. The PROPER was validated for air flow, ISO class cleanliness level, oxygen and carbon-dioxide gas concentrations during exhalation.

**Figure:**
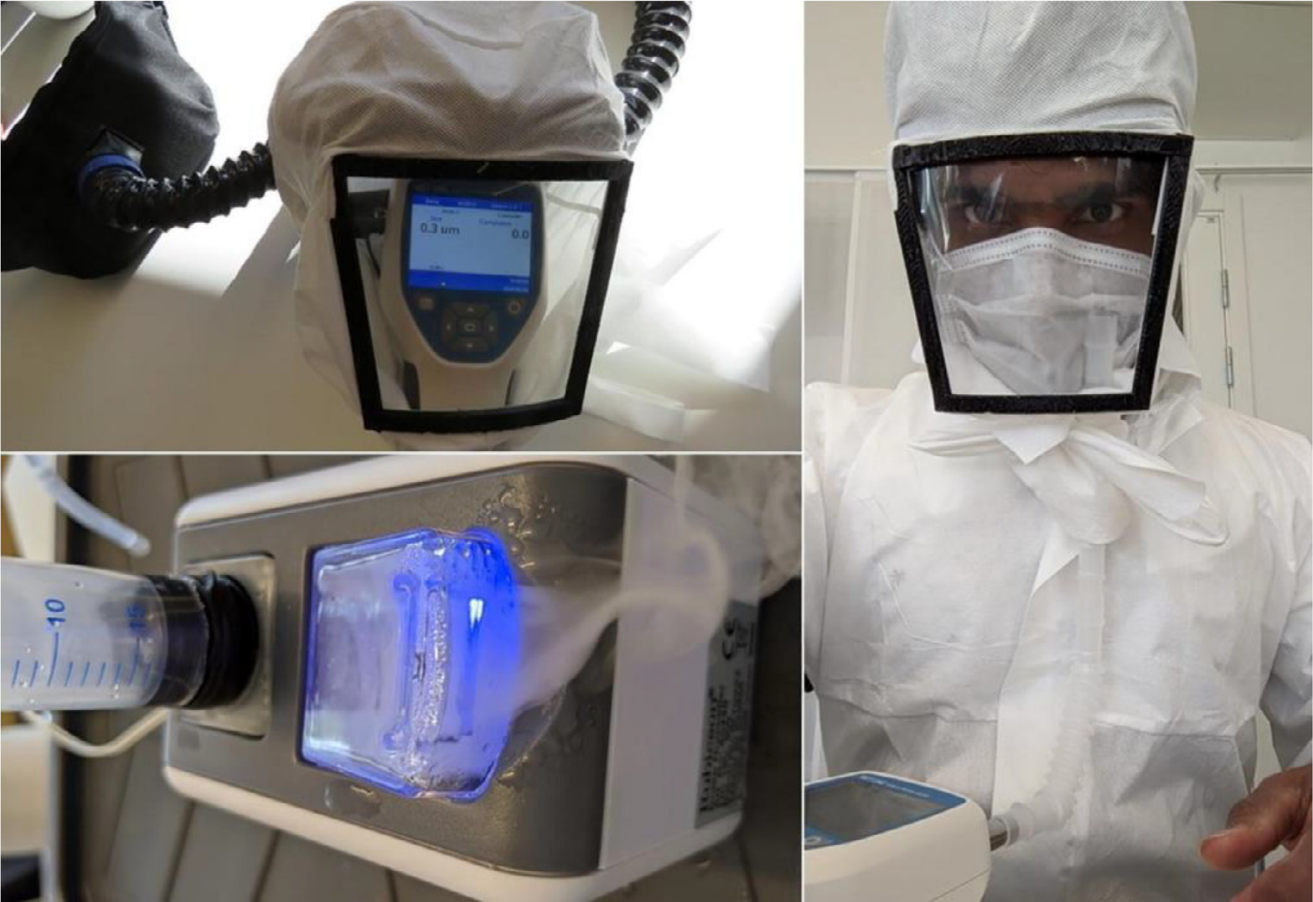

In addition to protecting people from infection, it was also important to determine who is infected. On this front, Abuzairi and colleagues from Indonesia developed the *iThermowall, which is an infrared thermometer on the wall to be used for fever screening* in public areas without an operator.

**Figure:**
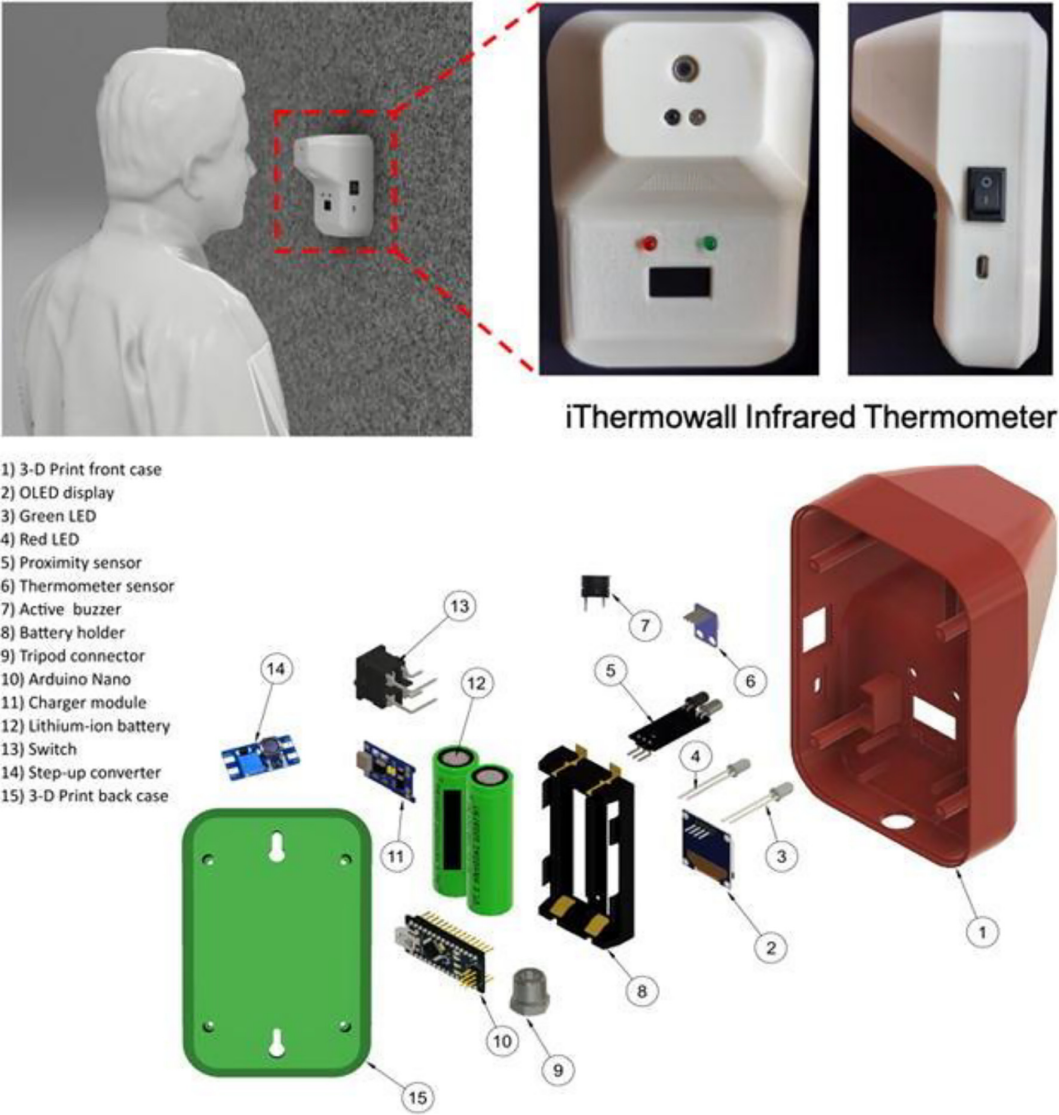

In other regions of the world the bottleneck for COVID-19 testing was the availability of the humble nasal swab. A major effort throughout the world went into making 3-D printable swabs primarily with high-end SLA 3-D printers. Gallup et al. in the U.S. developed a *parametric nasopharyngeal swab* for sampling COVID-19 that was in two components (a head with engineered break point and various handles), which minimized print time on relatively slow SLA printers, enabled the use of small volume consumer grade SLA printers, and enabled production of handle on more accessible material extrusion-based 3-D printers.

**Figure:**
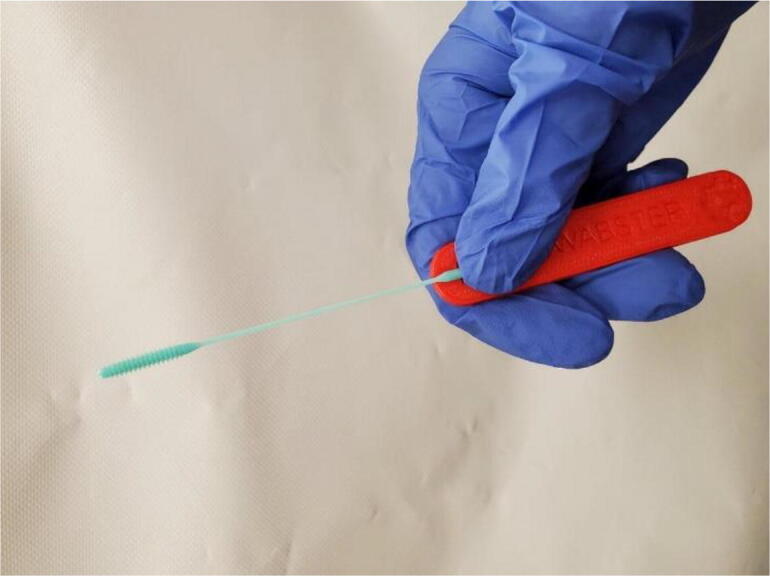

Lastly, for those infected that had serious issues ventilators were needed (luckily much less often than originally feared). Mora and a team in Brazil and MIT, developed a *taxonomy of the many open source hardware mechanical ventilators* that were developed. Other teams like those from Europe developed new open source ventilators like the *ATMO-vent* and in the U.S. a *partially RepRapable automated open source bag valve mask-based ventilator*. Together these open hardware designs, make the entire global community much more resilient to future pandemics as many of the *devices and products needed for this pandemic have now been designed and vetted*. With these designs freely available and ready for digital distributed manufacturing we will be much better prepared for the next pandemic. Lastly, and perhaps most importantly the pandemic has demonstrated the efficacy of the open hardware model applied to not only rapid prototyping of new designs but also distributed digital manufacturing. As we have now well established when focusing on scientific devices, the costs are substantially lower. In a review I completed last year there was overwhelming evidence for a wide range of scientific tools, that *open source technologies provide economic savings of 87% compared to equivalent or lesser proprietary tools*. It is now clear that we should start prioritizing funding for development of open source medical as well as scientific hardware for the benefit of the global community.

**Figure:**
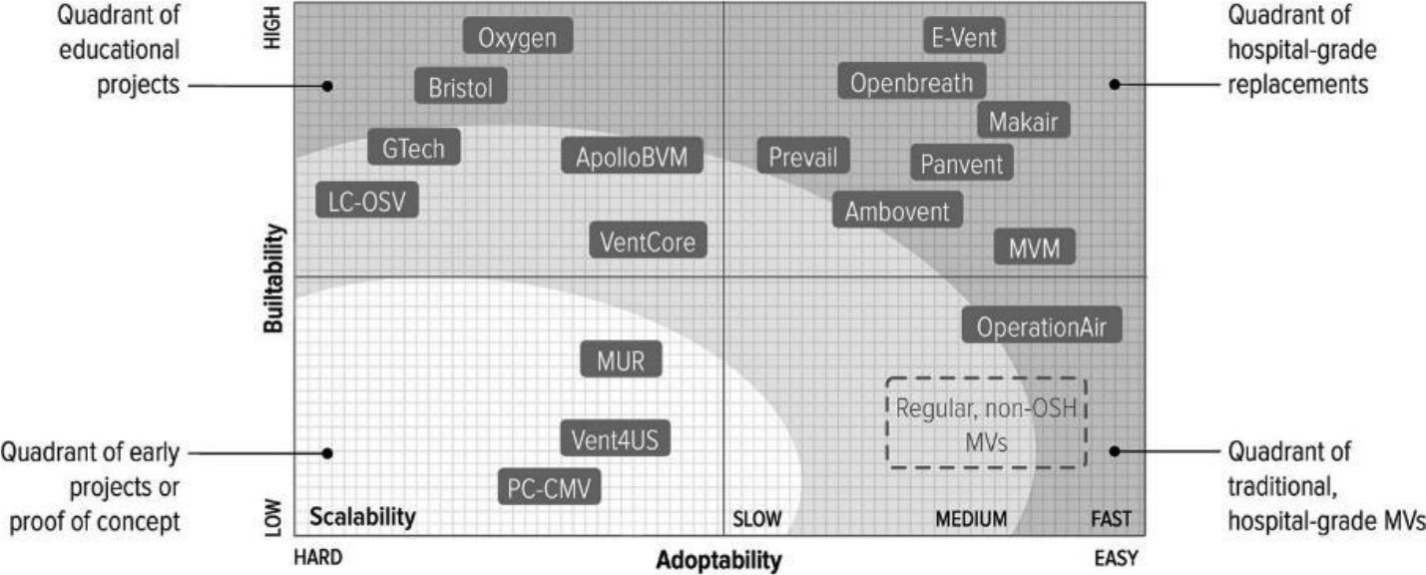

To read about these or any of the other technologies in the *HardwareX* Special Issue: COVID-19 Medical Hardware please see: https://www.sciencedirect.com/journal/hardwarex/special-issue/1037TX0Z2W8.

